# Monitoring the Simultaneous Implantation of Ti and Tb Cations to a Sacrificial Template and the Sol-Gel Synthesis of Tb-Doped TiO_2_ (Anatase) Hollow Spheres and Their Transition to Rutile Phase

**DOI:** 10.3390/ijms232113162

**Published:** 2022-10-29

**Authors:** María Teresa Colomer, Florencia Vattier

**Affiliations:** 1 Instituto de Cerámica y Vidrio (ICV), Consejo Superior de Investigaciones Científicas (CSIC), c/Kelsen 5, E-28049 Madrid, Spain; 2 Instituto de Ciencia de Materiales de Sevilla (ICMS), Consejo Superior de Investigaciones Científicas (CSIC)-Universidad de Sevilla, c/Américo Vespucio 49, E-41092 Sevilla, Spain

**Keywords:** hollow sphere, Tb, TiO_2_, implantation, sol-gel, X-ray methods

## Abstract

Tb-doped TiO_2_ (anatase) micro-hollow spheres (HSs) with nano-shells, in the range 0.00–3.00 at.% Tb, were successfully synthesized by a simultaneous chemical implantation route of both Ti and Tb cations from chlorides to a poly-styrene (PST)-co-poly-divinyl benzene (PDVB) sacrificial template, followed by controlled hydrolysis and polycondensation reactions. After water addition to the mixture of the precursors with the template, a decrease in the intensity and a shift to lower wavenumbers of the C=O absorption band in the IR spectra can indicate not only the anchoring of Ti and Tb ions to the carbonyl group of the template but also the hydrolysis of the implanted precursors. This latter process can involve a proton attack on the Ti–Cl, Tb–Cl and C=O bonds, the occupation of a vacant site by a water molecule, and then the dissociation of the dangling Ti–Cl, Tb–Cl ligands and C=O bonds. It gives rise to Ti_1−x_Tb_x_[(OH)_4−u_Cl_v_]@PST–PDVB and Ti_1−x_Tb_x_[(OH)_4−y_]@PST–PDVB complexes (x = 0.00, 0.0012, 0.0170 and 0.030). Finally, polycondensation of these species leads to Ti_1−x_Tb_x_O_2−w′_@PST–PDVB compounds. After subsequent thermal removal at 550 °C of the template, the IR bands of the core (template) totally vanished and new bands were observed in the 400–900 cm^−1^ region which can be attributed to the metalloxane bondings (M–O, M’–O, M–O–M, M–O–M’ and/or M’–O–M’, being M and M’ = Ti and Tb, respectively, i.e., mainly vibration modes of anatase). Then, micron-sized HSs of TiO_2_ and Tb-doped-TiO_2_ (anatase) were obtained with nano-shells according to field emission gun scanning electron microscopy (FEG-SEM) and transmission electron microscopy (TEM) observations. Furthermore, X-ray photoelectron spectroscopy (XPS) measurements confirmed the presence of Tb^4+^ (38.5 and 41.2% for 1.70 and 3.00 at.% Tb, respectively) in addition to Tb^3+^ in the resulting HSs, with increasing Tb^4+^ content with both Tb doping and higher calcination temperatures. Then, these HSs can be considered as rare earth (RE) co-doped systems, at least for 1.70 and 3.00 at.% Tb contents being the transition to rutile phase favored by Tb doping for those compositions. Finally, diffusion of Tb from the inner parts to the surface of the HSs with the calcination treatments was also observed by XPS.

## 1. Introduction

Titanium dioxide has excellent physico-chemical properties and unique applications in photocatalysis, gas sensors, solar cells, etc. Extensive research studies have been focused on controlling the microstructure, size and morphology of TiO_2_ to achieve novel and enhanced properties [1,2,3,4]. In particular, TiO_2_ HSs attract great interest due to their low density, higher surface area, better separation of electrons and holes, surface permeability, etc. For those reasons, the synthesis of RE-doped TiO_2_ materials as HSs with improved photoluminescent and/or photocatalytic properties is in focus [5,6,7,8]. From a simple point of view, hollow micro-nanospheres can be easily fabricated through digging void space inside solid spheres. According to the preparation process and formation mechanism three major methods are the most widely used to prepare hollow structures: (i) hard template, (ii) soft template, and (iii) self-template method. Among all three commonly employed protocols the first strategy has been extensively adopted in the fabrication of hollow micro and nanospheres as it is straightforward to use. This route is the approach considered by many researchers to be the most robust and reliable pathway to date for the preparation of hollow particles with a well-defined shape, size, and particle-size distribution in relatively large quantities [9,10,11]. This method is based on the implantation of a metal ion from a metalorganic compound or an anhydrous salt dissolved in a nonpolar solvent to penetrate the template surface and locate preferentially underneath the surface [10]. Hollow particles can be obtained by subsequent removal of the template by calcination in air. This type of synthesis has been used in previous works to obtain HSs of different compositions (e.g., Al_2_O_3_ [10], TiO_2_ [11] and In_2_O_3_ [12]) and also for the synthesis of RE-doped anatase and rutile HSs such as Er-doped TiO_2_ [5] and Tb-doped TiO_2_ HSs [7]. In this study, a simultaneous chemical implantation of the two cations has been established with the aim of developing and optimizing the synthesis of the Tb-doped anatase HSs. In previous works where two cations are needed Ti^4+^, the major cation is first implanted to the sacrificial template and the RE cation is added to the suspension obtained after implantation of the major cation [5,7]. In this research, we have used for the first time to the best of our knowledge a simultaneous chemical implantation of both cations not only the major one. In this way, a better Tb distribution in a faster and more easily conducted procedure is expected to be achieved. After implantation controlled hydrolysis was performed. In addition, it is also the first time to the best of our knowledge that the reactions that take place during this type of HSs synthesis have been monitored. Furthermore, it will allow us the control of the reaction times and to extend this type of synthesis to any type of doped TiO_2_ HSs. Then, the present work aims at monitoring the steps that occur during the implantation of the cations to the polymer and during the sol-gel synthesis of Tb-doped TiO_2_ (anatase) HSs through the preparation of Ti_1−x_Tb_x_O_2−w__′_@PST–PDVB core-shells. We have studied the formation process, the morphology, the texture, the structure, the chemical composition, and the thermal evolution of the as-obtained micro-nano TiO_2_ and Tb-doped TiO_2_ HSs. Furthermore, the anatase–rutile transition (ART) in these types of materials is also studied and discussed since controversial results have been found regarding the enhancement or the inhibition of this transformation in the previous literature of Tb-doped TiO_2_ materials [13,14,15,16].

## 2. Results and Discussion

### 2.1. Monitoring Ti and Tb Simultaneous Implantation and Sol-Gel Synthesis of Ti[(OH)_4−y_Cl_y_]@PST–PDVB, Ti_1−x_Tb_x_[(OH)_4−u_Cl_v_]@PST–PDVB, TiO_2_ and (Ti_,_Tb)O_2−w__′_ HSs (IR, FEG-SEM-XEDS and XPS)

A PST–PDVB copolymer was chosen as a sacrificial template since both anhydrous chlorides, i.e., TiCl_4_ and TbCl_3_ employed in the synthesis can be incorporated to the copolymer via the formation of a coordination bond between each chloride and the PST and/or the PDVB polymers. It is due to the ability of chlorides to accept PST moiety and the π electrons donated from the electron rich PDBV. It can lead to PST–PDVB–TiCl_4_ [17], PST–PDVB–TbCl_3_ and/or PST–PDVB–TiCl_4_–TbCl_3_ complexes.

**Figure 1:**
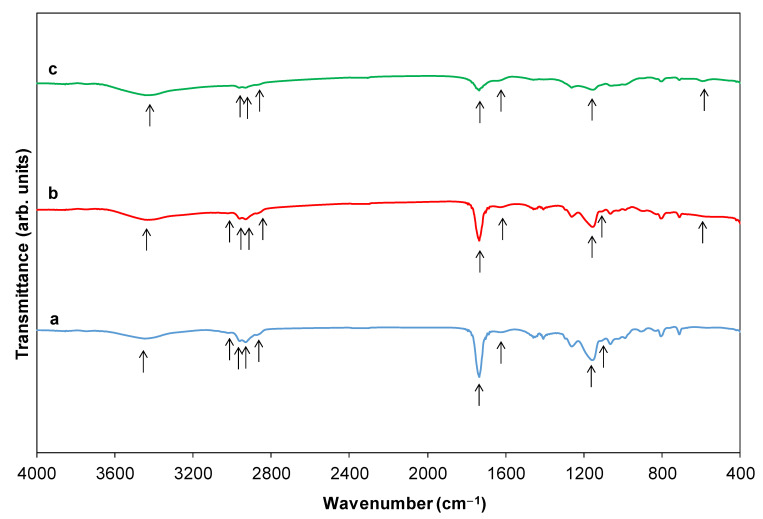
Normalized IR spectra of the template (a), of the template + TiCl_4_ and TbCl_3_ mixture + HCl_aq_ after 2 h of reaction (b) and after 5 h of reaction (c), i.e., of the Ti_1−x_Tb_x_[(OH)_4−u_Cl_v_]@PST–PDVB intermediate compound (Tb content in (b) and (c) is 0.12 at.%). Arrows are added in order to follow the evolution of the IR bands being the related reactions explained in the text.

**Figure 2:**
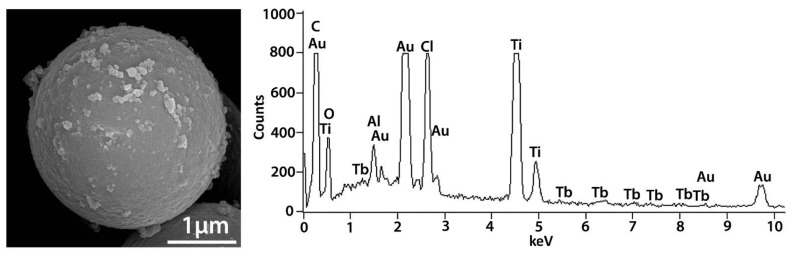
FEG-SEM picture of the mixture of the template + TiCl_4_ and TbCl_3_ precursors together with its characteristic XEDS analysis before the water addition, i.e., before hydrolysis and polycondensation reactions (HSs with 0.12 at.% Tb).

The template used here shows as its main features the following bands (Figure 1a): a slight band centered at 3446 cm^−1^ and one at approximately 1623 cm^−1^ (they are indicated with arrows in Figure 1a), corresponding to the stretching and bending vibration of hydroxyl groups, respectively [18]. Peaks in the region of 1640–1680 cm^−1^ are related to C=C vibrations and those in the region of 2873–2960 cm^−1^ are associated with =C–H bonds. The band at 2964 cm^−1^ can correspond to the stretching and antisymmetric vibration of the C–H in the carbonyl group. In addition, the characteristic absorption peak at 1735 cm^−1^ is associated with a C=O stretching vibration of an ester (it is indicated with an arrow in Figure 1a) [19]. The bands at 1262 and 2930 cm^−1^ are also attributed to the stretching and antisymmetric vibrations, respectively, in the carbonyl group of the C–H [20]. The spectrum also shows strong intensity absorption at 1163 cm^−1^ associated with a C–O bond, suggesting a carboxilyc acid ester that has been grafted with the PDVB polymer (it is indicated with an arrow in Figure 1a). The peaks observed at 1616 and 901 cm^−1^ can be a result of the conjugation of the aromatic ring present in the DVB molecule. The bands at the 700–870 cm^−1^ range are attributed to the aromatic C–H out-of-plane bending vibrations and absorption bands of unsubstituted styryl residues [18]. The presence of para-crosslinks from the PDVB polymer is demonstrated by the peak at 840 cm^−1^. Moreover, the band at 712 cm^−1^ (it is indicated with an arrow in Figure 1a) can be attributed to the skeletal C–C stretching vibration of tri-substituted benzene rings [21]. Furthermore, in the IR of Figure 1a, one characteristic absorption band of mono-substituted vinyl groups can be also recognized (900 cm^−1^), likely due to unreacted double bonds during the polymerization reaction. Those of the vinyl aromatic bonds at 1440 and 1490 cm^−1^ and those that match the skeletal vibration at 1603 cm^−1^ can be also detected in the spectrum. In addition, the surface of the pure template contains an appreciable amount of complex functional groups, including hydroxyl and carbonyl groups (Figure 1a), which provide ideal binding sites for the chlorides. After addition of the chlorides and before the water was added to the mixture (template + chlorides), any new peak is observed over the examined wavenumbers by IR. However, shrinkage (4.2%) of the average diameter size of the template after mixing with the TiCl_4_ or TiCl_4_ + TbCl_3_ mixture is detected by FEG-SEM (please compare Figure S1 and Figure 2). This fact, together with the color change of the template particles from white to light brown, suggests that a reaction between the chloride/s and the spacer occurs before hydrolysis. However, Tb is not observed by XPS even when the surface of the spheres was etched with Ar ions at 10^−6^ torr, 5 KV and 10 mA, during 50 min. It may be because Tb is located underneath the surface of the template and with these etching conditions is not observable. However, the presence of Tb is confirmed by FEG-SEM-XEDS (Figure 2). Those analyses also showed a homogeneous distribution of Tb on the HSs.

After the addition of water, IR spectra revealed a decrease of the intensity of the bands from 500 to 800 cm^−1^ for benzene ring C–H bending, indicating the donor–acceptor interaction of carbon–carbon (π) bond on the benzene rings with TiCl_4_ and TbCl_3_ (Figure 1a,b). A decrease wavenumber of 5 cm^−1^, when compared with the value of the band for the pristine template, i.e., 1740 vs. 1735 cm^−1^ and a shift of the C=O absorption band in the IR spectra occur. Both facts can be associated with the anchoring of Tb and Ti ions to the carbonyl group (Figure 1b,c). The band intensity centered at 3025 cm^−1^ (=CH– stretching of benzene ring) decreases (marked with an arrow in Figure 1a–c), indicating a reduction in the electron density on the carbon–carbon (π) double conjugated bond. This can be due to the donation of electron density from π bond to the vacant d orbital of Ti^4+^ in TiCl_4_ and partial vacant f orbital of Tb^3+^ in TbCl_3_. Furthermore, those bands related to the stretching and antisymmetric vibration of the C–H in the carbonyl group (2873–2960 cm^−1^) also decreased [18] (Figure 1b,c) compared with the bands of the spectrum for the template (Figure 1a). Both facts indicate not only that the carbonyl group of the template can have some kind of interaction with the chlorides and with Ti_1−x_Tb_x_[(OH)_4−u_Cl_v_]^δ^^+^ and Ti_1−x_Tb_x_[(OH)_4−y_]^ρ+^ species [18] but also that the carbonyl group takes part during the hydrolysis process as an electron donor for the H_3_O^+^ (electrophile attack). Furthermore, the bands in the range 1150–1085 cm^−1^ of the template (C–O stretching and bending vibrations) [18] also decreased with the hydrolysis process (Figure 1a–c). A new band at 615 cm^−1^ (Figure 1b,c) is also noted which can imply the formation of metalloxane bonds (it is marked with an arrow in Figure 1b,c).

Then, the IR spectra indicate that the hydrolysis reaction mechanism involves proton attack on the Ti–Cl, Tb–Cl and C=O bonds, the occupation of a vacant site by a water molecule, and then the dissociation of the dangling Ti–Cl, Tb–Cl ligands and C=O bonds.

**Figure 3:**
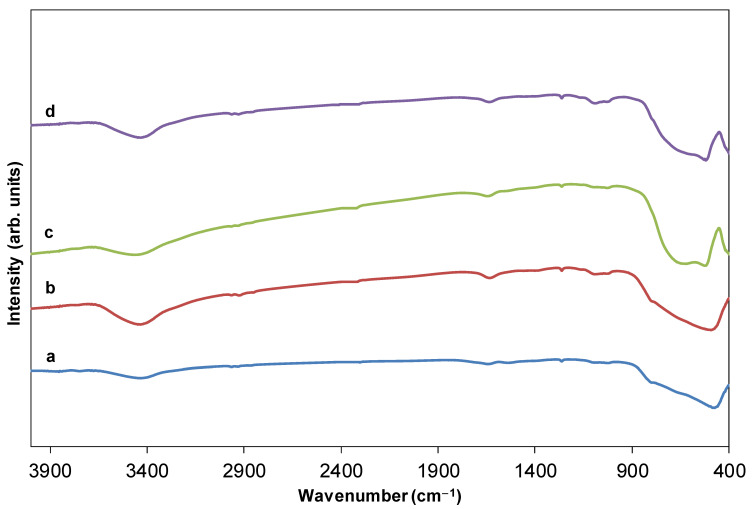
Normalized IR spectra of 0.00%_550 and 1.70%_550 (a) and (b), respectively, and 0.00%_1000 and 1.70%_1000 (c) and (d), respectively.

For samples calcined at 550 °C and greater, the IR bands of the template totally vanished (Figure 3a–d). Moreover, new bands were observed in the 400–900 cm^−1^ region that can be attributed to the metalloxane bondings (M–O, M’–O, M–O–M, M–O–M’, and/or M’–O–M’, being M and M’ = Ti and Tb, respectively), i.e., mainly vibration modes of anatase (Figure 3a,b). In particular, the band centered at 480 cm^−1^ is observed for both undoped and doped HSs that can be attributed to the transverse optical vibrations of the Ti–O bonds [22,23,24] in anatase. Only bands of rutile are observed for HSs calcined at 1000 °C (523 and 661 cm^−1^ for the undoped, and 520 and 658 cm^−1^ for the doped ones) (Figure 3c,d, respectively). The shape of these bands varied in accordance with the different crystal phases; anatase has less-defined Ti–O stretching bands as it possesses a less ordered structure (Figure 3a,b) compared to rutile (Figure 3c,d), respectively. For the HSs calcined at 800 °C vibration modes of both anatase and rutile are observed (not shown here) in accordance with XRD results (see below in Section 2.5).

**Figure 4:**
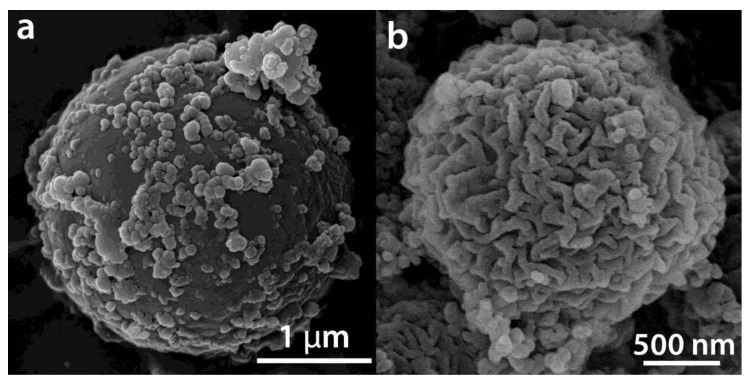
FEG-SEM pictures of the template + TiCl_4_ and TbCl_3_ mixture + HCl_aq_ after 5 h of reaction (**a**) (it corresponds to the sample of IR in Figure 1c) and FEG-SEM picture of the 0.12 at.% Tb HSs after 7 h of reaction and calcination at 550 °C (0.12%_550) (**b**).

**Figure 5:**
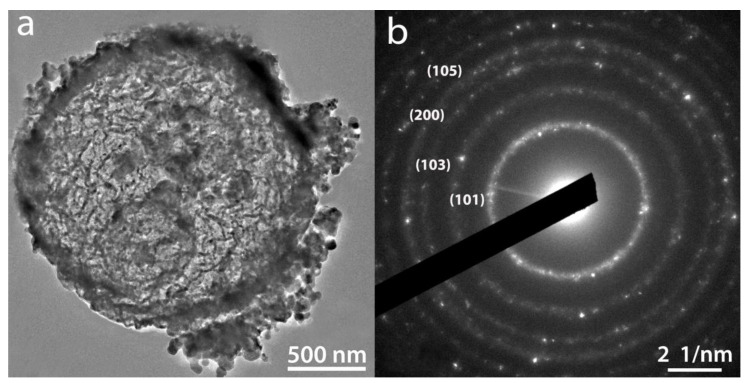
TEM picture of a 0.12%_550 HS where the shell thickness of the capsule can be observed (**a**). SAED pattern corresponding to the HS is shown in (**b**).

After hydrolysis and polycondensation, another color change from light brown to dark brown is observed for the particles. Figure 4 shows FEG-SEM pictures of the template + TiCl_4_ and TbCl_3_ mixture + HCl (aq) after 5 h of reaction for the 0.12 at.% Tb sample (a) and the same HSs after 7 h of reaction and calcination at 550 °C for 30 min (b). The presence of Cl^−^ in the intermediate compounds before washing and calcination as well as the presence of both Ti and Tb was confirmed by XEDS (Figure S2). Figure 4b shows a uniform hollow Tb-doped TiO_2_ microsphere with an outer average diameter size of 1.8 ± 0.2 µm, confirmed by TEM (Figure 5a). The size of the spheres after calcination at 550 °C suffers a shrinkage process of approximately 37% when compared with uncalcined spheres. A rough surface is noted related to the presence of aggregates of nanoparticles. In addition, the average shell thickness of the HSs is 64.0 ± 0.2 nm (Figure 5a). The SAED diagram shown in Figure 5b provides a direct evidence of the structure of the material which corresponds to anatase phase according to XRD results (see below in Section 2.2.)

### 2.2. Texture and Structure of Ti[(OH)_4−y_Cl_y_]@PST–PDVB, Ti_1−x_Tb_x_[(OH)_4−u_Cl_v_]@PST–PDVB, TiO_2_ and (Ti_,_Tb)O_2−w__′_

**Figure 6:**
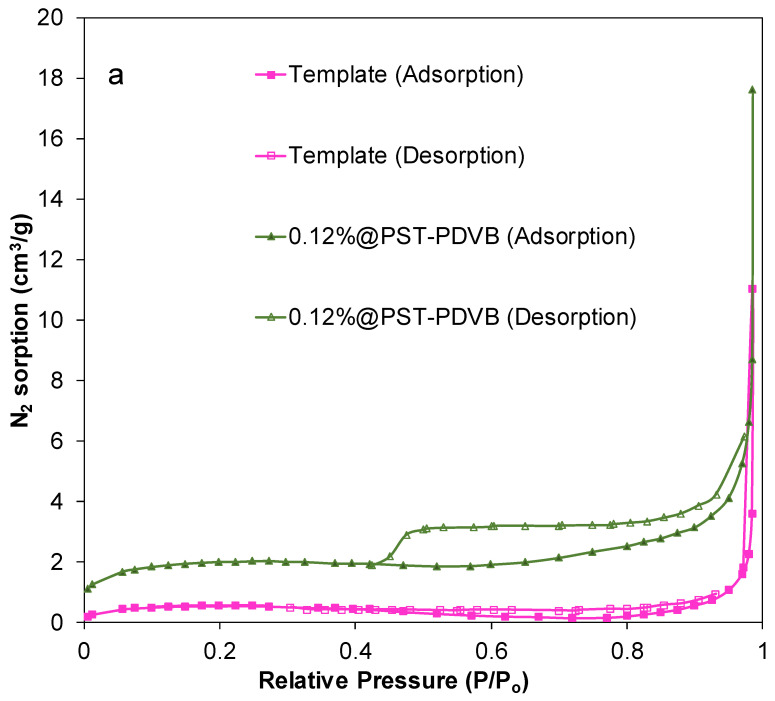

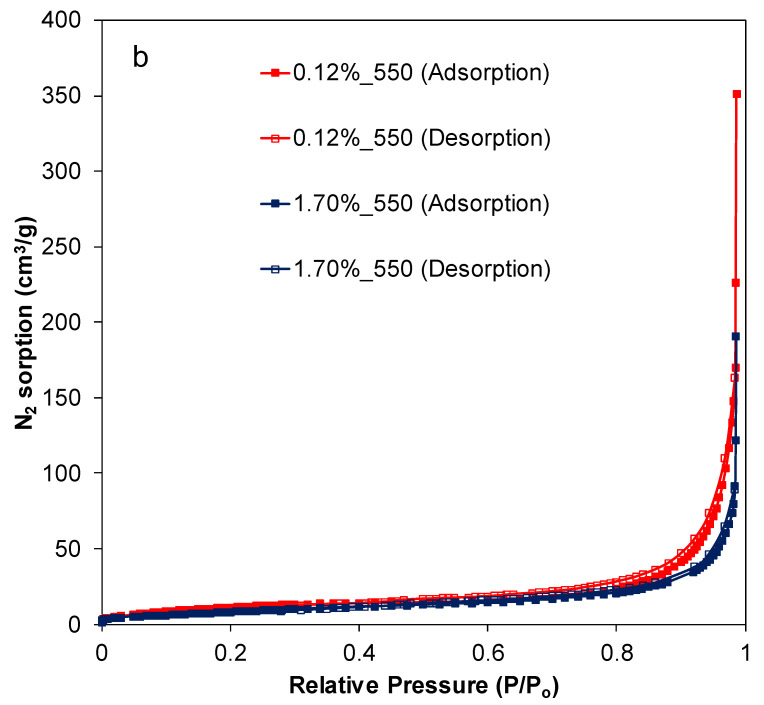
(**a**) N_2_ adsorption–desorption isotherm curves of the template and of the Ti_0.988_Tb_0.012_ [(OH)_4−u_Cl_v_]@PST–PDVB composite. (**b**) N_2_ adsorption–desorption isotherm curves of 0.12%_550 HSs and 1.70%_550.

The HSs were analyzed by a LECO analyzer in order to check whether residual C and N are present in the samples after calcination at 550 °C for 30 min. These analyses indicated that the HSs are free of both C and N. Figure 6a shows the N_2_ gas adsorption–desorption isotherms of the template and the intermediate compound obtained at the end of the polycondensation reaction and before calcination, i.e., of Ti_0.988_Tb_0.12_[(OH)_4−u_Cl_v_]@PST–PDVB intermediate product. The template is a non-porous solid according to its isotherm (Figure 6a). A slight hysteresis in the adsorption–desorption cycle found can indicate non-rigid aggregates of plate-like particles or a pore network of macropores between the spheres [25]. In this case, the latter option is the plausible one. In addition, the N_2_ gas adsorption–desorption isotherms of the Ti(OH)_4_@PST–PDVB and Ti_1−x_Tb_x_[(OH)_4−u_Cl_v_]@PST–PDVB composites are characteristic of mesoporous materials. In particular, their isotherms correspond to type IV with a loop of type H2 according to the IUPAC classification [25]. Figure 6b shows the N_2_ gas adsorption–desorption isotherms of two calcined Tb-doped TiO_2_ capsules (1.70 and 3.00 at.% Tb) after their calcination at 550 °C for 30 min. A very slight hysteresis in the adsorption–desorption cycles found for these calcined spheres can indicate a pore network of macropores between HSs [25], as in the case of the template. The specific surface areas found ranged from 44 ± 3 m^2^/g for the undoped to ~34 ± 4 m^2^/g for doped HSs calcined at 550 °C for 30 min, respectively (Table 1). After calcination of the HSs at 1000 °C, the specific surface areas found were approximately 0.1 ± 0.5 m^2^/g.

**Figure 7:**
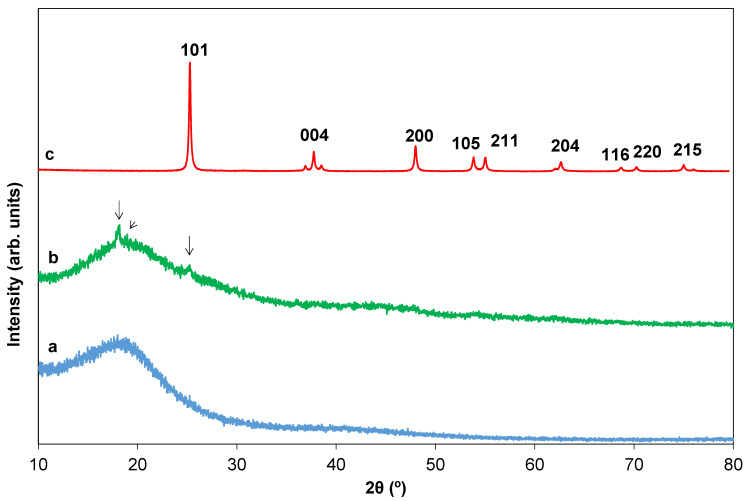
Indexed XRD patterns of the template (a), the Ti_1−x_Tb_x_[(OH)_4−u_Cl_v_]@PST–PDVB (x = 0.012) intermediate product after 5 h of reaction (b) and Ti_1−x_Tb_x_O_2−w′_ (x = 0.012) after calcination at 550 °C for 30 min (0.12%_550) (normalized XRD pattern) (c). The peaks at 18.18, 18.74 and 25.26° that can confirm the implantation of TiCl_4_ and TbCl_3_ in the PST–PDVB matrix are indicated with arrows.

Figure 7 shows the XRD diffraction patterns of the template (a) after the simultaneous implantation of both TiCl_4_ and TbCl_3_ (0.12 at.% Tb) to the carbonyl groups of the template, followed by the addition of HCl_aq_, i.e., when the hydrolysis and polycondensation reactions are taking place (b), and after calcination at 550 °C for 30 min (c). The virgin template exhibited a broad diffraction band at 2θ of the range 15.00–30.00° suggesting the amorphous structure of the polymeric scaffold (Figure 7a). However, in the case of the intermediate product formed after implantation and the partial hydrolysis and polycondensation reactions (Figure 7b), the XRD pattern shows a type of semi-crystalline structure in which the intensity of the characteristic reflection band at 2θ = 15.00–30.00° has diminished without altering its position. The reason for the decrease in intensity is likely due to the accommodation of TiCl_4_ and TbCl_3_ molecules in the PST–PDVB matrix forming complexes with the template. In addition, new peaks at 18.18, 18.74 and 25.26° (indicated with arrows) could confirm the implantation of both chlorides in the polystyrene matrix (Figure 7b). In fact, in a previous work, Rahmatpour et al. [17] found a peak at 2θ = 18.06° that assigned to the implantation of TiCl_4_ onto the PST–PDVB which is in good agreement with that found in this study (18.18°). After calcination at 550 °C for 30 min pure anatase phase was completely crystallized for all compositions (Figure 7c is shown as a way of example). Neither brookite nor rutile phases were noted for any composition calcined at that temperature. In addition, neither metal clusters of Tb nor impurity phases such as Tb oxides were detected.

### 2.3. Thermal Evolution of the Ti_1−x_Tb_x_[(OH)_4−u_Cl_v_]@PST–PDVB followed by DTA-TG Analysis

**Figure 8:**
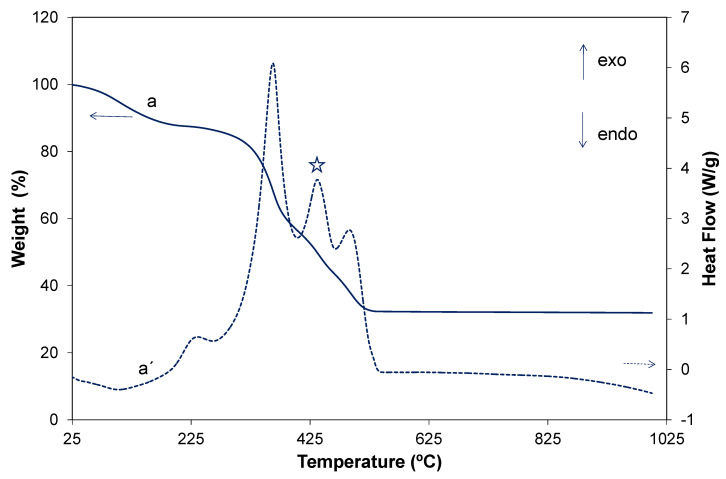
Thermogravimetric and differential thermal analyses curves of the Ti_0.988_Tb_0.012_ [(OH)_4−u_Cl_v_]@PST–PDVB composite (a and a′, respectively) in air (Tb content is 0.12 at.%). The star indicates the exothermic peak related to Cl^−^ removal.

The thermal behavior of the PST–PDVB was analyzed in a previous work [10]. The behavior of the Ti_1−x_Tb_x_[(OH)_4−u_Cl_v_]@PST–PDVB composite obtained at the end of the hydrolysis and polycondensation reactions and before washing was studied by DTA-TG in air (Figure 8). The TG curve of the template displays, as was observed before [10], a weight loss of 100% during the calcination process, which is the burning of the spheres. According to those and in line with our DTA-TG results the sacrificial template begins to degrade at temperatures above 250 °C and shows two main weight loss regions. At the temperature range of 300–400 °C, a pronounced exothermic peak occurs in accordance with a significant weight loss of approximately 60%. This is due to the oxidation of the low melting, pendant alkyl groups in the template polymers. A second exothermic peak with a weight loss of approximately 40% follows at the temperature region 450–530 °C. This resulted from the decomposition of the aromatic rings [26]. The TG curve of the Ti_1−x_Tb_x_[(OH)_4−u_Cl_v_]@PST–PDVB composite shows four loss stages: below 200 °C a weight loss of 12.2 wt.% can be due to the dehydration of the microspheres and OH^−^ and the physisorbed volatile organic removal. This weight loss is accompanied by an endothermic peak centered at 99.3 °C that is not observed for the free template. Four pronounced exothermic peaks occur at 232.2, 365.8, 436.0 and 484.4 °C in accordance with four significant weight losses of a total amount of approximately 55.4 wt.%. The first, second and fourth peaks can be associated with the decomposition, oxidation and burning of the template, respectively [10], as is indicated above. The third peak detected at 436.0 °C and indicated with a star in Figure 8 is related to a weight loss that can be due to residual Cl^−^ elimination [27]. The presence of Cl^−^ was also confirmed by FEG-SEM-XEDS, as is mentioned above. The percentage of residual weight is approximately 32.0%, which accounts for the final (Ti,Tb)O_2−w′_ product, indicating that the HSs were prepared in a considerably high yield by this method. We observed that the degradation temperature of the polymer slightly increases when the polymer is part of the Ti_1−x_Tb_x_O_2−z__′_@PST–PDVB composite, i.e., a shifting in the temperature events for both TG and DTA curves is detected for the composite with respect to the PST–PDVB template [10]. This is plausible since the polymer is covered by a coating of Ti_1−x_Tb_x_[(OH)_4−u_Cl_v_] which slightly hinders the calcination of the template.

### 2.4. Compositional Analysis of the Calcined HSs by XPS

The nature and composition of the HSs surfaces were analyzed in selected samples by XPS. As is mentioned above, in the analysis of the HSs before calcination, Tb was not detected for any sample unlike in the calcined HSs. The survey spectra and the spectral regions of Tb 3d, Ti 2p and O 1s peaks for the samples calcined at 550, 800 and 1000 °C are depicted in Figure S3. The Ti 2p photoemission signals consist in a doublet at approximately 465 and 459 eV which can be attributed to Ti 2p_1/2_ and Ti 2p_3/2_, respectively. These photoemission signals are separated by 5.8–5.7 eV according to the values of ΔBE reported for Ti^4+^ in TiO_2_ nanoparticles with both anatase and rutile structures [15,28,29]. As is expected for air-exposed samples they do not show any signal corresponding to Ti^3+^. The surface composition of the samples was obtained from the relative intensities of each element and they are collected in Table 2. As is mentioned above, since Tb content is very low for the sample with a nominal 0.12 at.% content, its determination is within the detection limit of the technique. For that reason, the spectrum of Tb is not deconvoluted.

**Figure 9:**
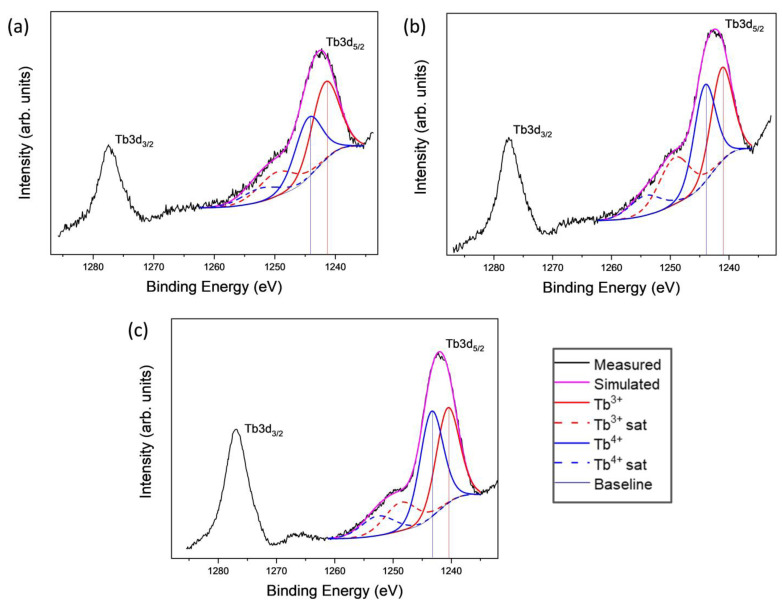
Experimental and fitted Tb 3d_5/2_ X-ray photoelectron spectral region for selected samples 3.00%_550 (**a**), 1.70%_800 (**b**), and 1.70%_1000 (**c**). Vertical lines are added for easy reading of the peak values on the X-axis.

The Tb 3d signal consists in a doublet for the two energetic levels Tb 3d_3/2_ and Tb 3d_5/2_ accompanied by characteristic satellite signals. Asymmetries in the peak shapes and satellite structures are well-characterized. By the analysis of high-resolution spectra it is seen that two chemical states of terbium, Tb^3+^ and Tb^4+^, are present [30,31,32]. Tb 3d_5/2_ spectra have been fitted with four components, i.e., an emission peak and one satellite signal for each oxidation state [7,33]. The fitting and the involved curves of the samples 3.00%_550, 1.70%_800 and 1.70%_1000 are shown in Figure 9. In all cases, the 3d_5/2_ photoemission peak for Tb^3+^ appears at approximately 1241 ± 0.4 eV and the corresponding satellite appears at 1249 ± 0.4 eV. Tb^4+^ oxidation state shows peaks at 1244 ± 0.5 and 1252 ± 0.6 eV for photoemission and the satellite signals, respectively. The quantification of these chemical states at the surface was possible from the relative intensities of the signals in the deconvoluted spectra; these data are shown in Table 3. The results indicate that when the samples were calcined at higher temperatures, a greater amount of Tb atoms migrate to the surface of the material from the inner part of the spheres. First, Tb is detected after calcination but not before and second, a larger Tb content is determined for a given composition as the calcination temperature increases. Diffusion of Tb to the surface with calcination temperature is then produced. The results also indicate that both a higher calcination temperature and a higher Tb content led to a larger Tb^4+^ content for a given composition in the first case and for a given temperature in the second. The oxidation of Tb^3+^ ions to attain the +4 valence state is due to the tendency of lanthanide ions to reach their most stable electronic configuration of a half-filled 4f shell. Then, as is mentioned above and according to our XPS results, HSs can be considered as RE co-doped systems, at least at higher Tb contents where Tb^3+^ and Tb^4+^ coexist and Tb^4+^ is present in a significant fraction.

The O 1s peaks are positioned at approximately 530.8 eV for all samples, the wide and asymmetric shape of high resolution spectra of the O 1s region indicate the presence of at least two different chemical states of oxygen [34,35]. The O 1s spectra have been decomposed into two contributions, i.e., the main contribution, at 530.7 eV, is attributed to Ti-O bonds in the TiO_2_ lattice [36,37], and the minor contribution, at 532.1 eV, corresponds to hydroxyl groups (Ti–OH) and adsorbed H_2_O [29]. Figure S4 shows the deconvolution of the signal fitting to two types of oxygen and the involved curves for the samples calcined at 550 °C (undoped, 1.70% and 3.00% Tb). The quantification of these results is presented in Table 3 ^(b)^.

### 2.5. Effect of Tb Doping and Temperature on the Structure and ART of the HSs

XRD studies were also performed in order to investigate the influence of both Tb content and thermal treatment temperature on the structure of the HSs. All HSs, undoped and doped calcined at 550 °C present anatase as unique phase. In Figure 7c, the XRD pattern of 0.12%_550 HSs is shown by way of example. In addition, anatase lattice parameters, cell volumes and crystallite sizes of the HSs calcined at 550 °C are collected in Table 4. The cell volumes of our samples are very similar to the undoped HSs and also among them independent of the Tb content. This can be due to the presence of a significant Tb^4+^ content in the anatase lattice as it has been proved by XPS. In addition, it must be also taken into account that Tb contents are very low in this study. An increment of the crystal size with Tb (Table 4) which, in turn can promote in principle the ART is also detected.

**Figure 10:**
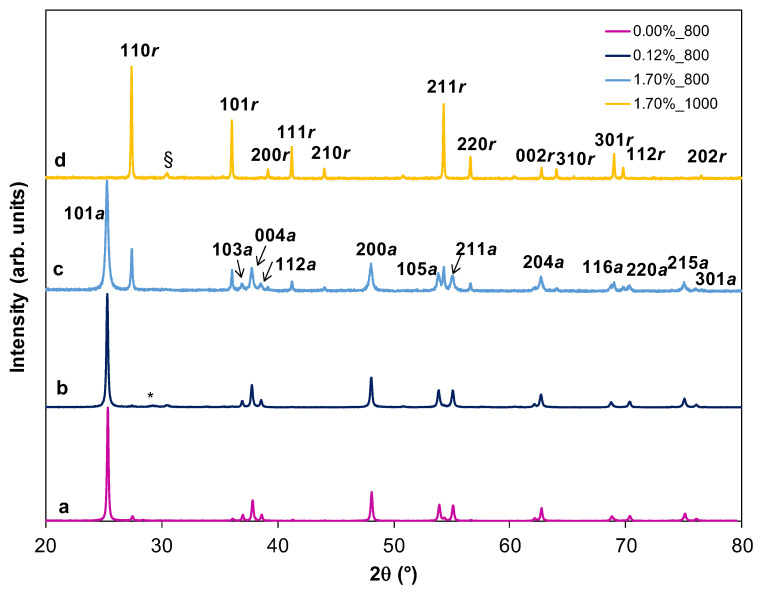
(a) Normalized and indexed XRD patterns of 0.00%_800 (a), 0.12%_800 (b), 1.70%_800 (c) and 1.70%_1000 (d). HSs. *a* indicates anatase and *r* rutile phase, respectively. The peak of the Si holder and SiO_2_ (contamination of agate mortar after milling: crystoballite quartz) are labeled * and §, respectively.

As is well known, ART is strongly dependent on the dopant content in TiO_2_ and on the calcination atmosphere [38]. ART in Tb-doped anatase materials is a controversial topic in the previous literature [13,14,15,16]. These discrepancies can be due to differences in the precursors used in the synthesis, the synthesis method itself, the firing schedule, the different atmospheres used during calcination, different morphologies of the titanium oxide, the presence of impurities, etc. These factors can lead to different crystallite sizes and different oxidation states in the final Tb-doped TiO_2_ powders. As is also well known, the different oxidation states influence the oxygen vacancies content for a given composition [38]. In general, cations of small size and valence > +3 accelerate the anatase–rutile transformation, while those with larger radii and valence lower than that of Ti^4+^ delay the transition. This is due to the electroneutrality condition, since to compensate for the different charge of the doping ion (+3) it is necessary to generate oxygen vacancies and/or interstitial Ti ions with an equal or lower valence. Therefore, if Tb is present as Tb^3+^ at Ti^4+^ sites, a delay of the transition should occur as for Er, Eu and Nd [5,39,40,41] doping. However, if an important fraction of Tb is as Tb^4+^, because of its smaller size (Tb^4+^ (VI) 0.76 Å) [42] the ART can be favored. In this study, according to XRD results, Tb seems to accelerate that transformation when Tb content is 1.70 or 3.00 at.%. It is observed that in the 0.00_800 the anatase–rutile transition is already initiated (Figure 10a). However, when doping content is 0.12 at.% Tb the presence of rutile is lower (2.0%) (Figure 10b) than in the former case (3.5%) (Figure 10a). In addition, for higher Tb contents rutile phase concentration increased (25.0% for 1.70_800) (Figure 10c). Then, Tb doping delays the ART when Tb is 0.12 at.%, while an enhancement of rutile content is observed at higher Tb contents. The delay produced for 0.12 at.% could be due to the presence of Tb only as Tb^3+^. This fact cannot be proved by XPS since Tb content is under the detection limit of this technique, as is mentioned above. At 1000 °C the HSs of all compositions are totally transformed into the rutile phase. Figure 10d shows 1.70 at.% Tb composition (1.70%_1000) as way of example.

The experimental results should be also analyzed considering the essential and stabilizing role of the entropy of mixing in the ART. Scoca et al. [16] claimed that an increase in the entropy of mixing when anatase is doped with Sm, Tb or Tm at low contents is produced and the ART is delayed due to the introduction of more and multiple quantum states, originated in vacancies and impurities in the anatase phase. This is due to possible arrangements of vacancies associated with oxygen bonded. However, further increase of RE doping over ~1.0–1.5 at.% prompted the ART. According to those authors [16] a larger number of impurities and/or dopants suggests that the increasing entropy of mixing could favor the stabilization of the metastable phase (anatase) because the −T∆S_mix_ term contributes to the minimization of the Gibbs free energy. Nevertheless, there are other effects that could invalidate this argument. For instance, increasing dopant content can prompt the ART by the formation of rutile nucleation centers. In fact, the results of Scoca et al. [16] show that co-doping at RE concentration ≥ 1 at.% destabilizes the anatase phase at lower annealing temperatures because of the aggregation of defects [16] and probably kinetic effects [43]. Our doped HSs, at least those doped with 1.70 and 3.00 at.% Tb, can be considered as co-doped systems given the amphoteric behavior of Tb. Then, at those Tb contents the ART is favored as is observed by Scoca et al. [16] in Tb-doped samples for Tb contents higher than 1 at.%.

**Figure 11:**
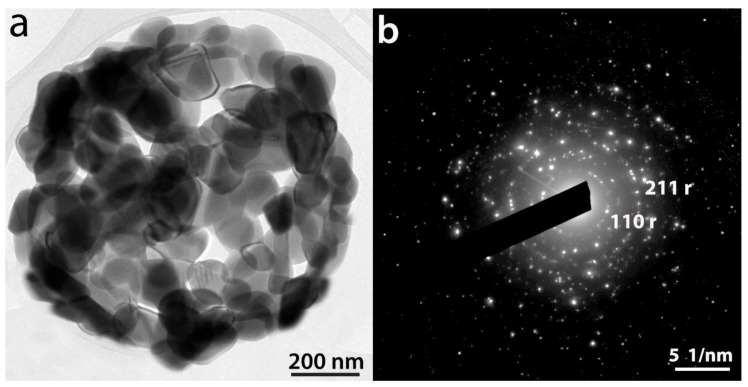
Representative TEM picture of a 1.70%_1000 HS (**a**) and SAED pattern corresponding to the HS is shown in (**b**).

Finally, Figure 11 shows a representative TEM picture of a 1.70%_1000 HS (a) and its corresponding SAED pattern (b). The electron diffraction pattern provides a direct evidence of the structure of the HS that corresponds to rutile phase according to XRD results (Figure 10d).

## 3. Materials and Methods

### 3.1. Synthesis of Ti[(OH)_4−y_Cl_y_]@PST–PDVB, Ti_1−x_Tb_x_[(OH)_4−u_Cl_v_]@PST–PDVB, TiO_2_ or (Ti,Tb)O_2−w__′_ HSs

A simultaneous chemical implantation of both cationic species, Ti and Tb, into the template was carried out, followed by the controlled hydrolysis and polycondensation reactions of both precursors. First, we obtain Ti_1−x_Tb_x_[(OH)_4−u_Cl_v_]@PST–PDVB core-shells using a PST–co–PDVB commercial nonporous template (Micropearl, SP-203, Sekisui Chemical Co., Osaka and Kyoto, Japan). The microspheres after washing with deionized water had an average size of 3.0 ± 0.1 µm in diameter (Figure S1).

The spacer was put into anhydrous heptane (99%, Sigma-Aldrich, Taufkirchen, Germany) and dispersed applying 2 min of ultrasound. Then, anhydrous TiCl_4_ (99.9%, Sigma-Aldrich, Taufkirchen, Germany) and anhydrous TbCl_3_ (99.99%; Sigma-Aldrich, Taufkirchen, Germany) in the case of doped materials, were both dissolved in anhydrous heptane prior to their simultaneous addition to the suspension formed by the template in heptane in order to achieve a simultaneous implantation of TiCl_4_ and TbCl_3_ at the surface of the polymer. After stirring and homogenization, HCl_aq_ (37%, Sigma-Aldrich, Taufkirchen, Germany) was added as catalyst and it included the water for the controlled hydrolysis of both chlorides. The system was introduced in a water bath at 75 °C for 7 h under stirring. After that, the product was centrifuged and washed with heptane twice. After each centrifugation, the powder was filtered and washed with heptane. Finally, it was put into the oven at 70 °C overnight. For the preparation of HSs, the calcination step used for removing the spacer was performed at 550 °C in air atmosphere with a dwell time of 30 min. In all cases, accumulative calcination treatments were performed after the initial calcination at 550 °C for 30 min in order to study the thermal evolution of the samples. The calcination temperatures employed were 750, 800 and 1000 °C for 30 min. All the systems had the same total volume and were obtained in a nominal atomic doping level Tb/Ti^4+^of 0.00, 0.12, 1.70 and 3.00 at.%. The samples after calcination are named as %Tb_calcination temperature; for example “3.00%_550” corresponds to a sample of 3.00 at.% Tb (nominal composition) calcined at 550 °C for 30 min.

### 3.2. Characterization Techniques

#### 3.2.1. IR

In order to confirm the chemical bonding structure and the formation mechanism of the Ti_1−x_Tb_x_[(OH)_4−u_Cl_v_]@PST–PDVB, Ti_1−x_Tb_x_[(OH)_4−u__′_]@PST–PDVB intermediate products, the (Ti,Tb)O_2−w__′_@PST–PDVB composite and the Tb-doped TiO_2_ HSs, IR spectra of the template—the reaction mixture at increasing times, at the end of the synthesis and finally, before and after the calcination steps—were performed. IR spectra of the suspensions were obtained between 4000 and 400 cm^−1^, after drying, at different times of hydrolysis and polycondensation reactions. In addition, IR spectra of the capsules were obtained before and after calcination. The amount of 0.8 mg of the sample was mixed with 300 mg of KBr before recording the spectra. The IR spectra were obtained with a PerkinElmer 1330 spectrophotometer (Waltham, MA, USA) with a 3600 data station, using the standard program CDS-13 for data handling.

#### 3.2.2. N_2_ Adsorption-Desorption

N_2_ adsorption–desorption isotherms, pore structure and specific surface area of the template, the core-shell structures and the hollow TiO_2_ and Tb-doped TiO_2_ spheres were determined from nitrogen gas sorption–desorption (ASAP 2020, Micromeritics Co., Norcross, GA, USA).

#### 3.2.3. DTA-TG Studies

Differential thermal analysis and thermogravimetry (DTA-TG) of the commercial template and of the as-synthesized powders before calcination at 550 °C for 30 min were performed in Perkin Elmer analyzers, DTA7 and TGA7 Models, respectively, (Waltham, MA, USA), using a typical sample size of 10 mg in a Pt crucible and a heating rate of 5 °C/min up to 1000 °C.

#### 3.2.4. C and N Analysis

After calcination at 550 °C for 30 min, C and N analysis were performed by a LECO analyzer (LECO CHNS-932 elemental analyzer, St. Joseph, MI, USA). The analyzer threshold value is 0.2% for each element.

#### 3.2.5. XRD Studies

Resulting powders were analyzed by X-ray powder diffraction in a diffractometer (X’Pert PRO, Panalytical, Malvern, UK) over the angular range 20–80° 2θ using Cu-Kα incident radiation and were used to confirm the crystalline phases present and their crystallite sizes. The crystalline phases of the powder samples were identified from the positions and intensities of the diffracted peaks using the ICDD database powder diffraction (http://www.icdd.com/). The files employed were the following: JCPDS-89-4921 for tetragonal TiO_2_-anatase phase, JCPDS-29-1360 for orthorhombic TiO_2_-brookite phase, and JCPDS-021-1276 for tetragonal TiO_2_-rutile phase, respectively. The lattice parameters of the samples were determined using a least-squares fitting method (Unitcell, Cambridge University, Cambridge, UK). The crystal size was calculated using the Scherrer equation [44]:

S = (Kλ)/(βcosθ)(1)

where K is the shape factor (0.9), λ is the X-ray wavelength (CuKα = 1.5405 Å), β is the full width at half maximum intensity (FWHM) in radians, and θ is the Bragg angle in degrees.

The anatase fraction was calculated from XRD data using the equation:

X_A_ = 1/[1+ 2.18 (A_R_/A_A_)] ± 2% (2)

where X_A_ is the molar fraction of anatase, and A_A_ and A_R_ are the total areas of the peaks of the X-ray intensities of the anatase and rutile strongest peaks, 101 and 110, respectively [45].

#### 3.2.6. XPS

XPS experiments were performed in a PHOIBOS-100 spectrometer (SPECS, Berlin, Germany) with a non-monochromatic Al Kα source (hν = 1486.6 eV) and the power of the X-ray source was 230 W (11.5 kV and 20 mA). The electron energy hemispherical analyzer was operated in the constant pass energy mode (SPECS PHOIBOS 100DLD). Low resolution survey spectra were obtained with a pass energy equal to 50 eV, whereas high energy resolution spectra of the main photoemission peaks from the detected elements (i.e., Tb 3d, Ti 2p, O 1s) were obtained with 35 eV as pass energy. The spectra were analyzed with the “CASA XPS” software, version 2.3.16.Dev52 (by Neal Fairly, UK). Shirley-type backgrounds were used to determine the areas under the peaks. The Tb 3d_5/2_ spectra were fitted with two components, for both Tb^3+^ and Tb^4+^, using Gaussian–Lorentzian functions (GL = 30). Each component was simulated by a main function with the same width (5.7–5.9 eV) and its associated satellite separated 2 eV and slightly wider (7.9 eV). Component positions and widths were kept constant in all fittings. The O 1s signal was fitted with two different components separated by 1.5 eV (GL = 30) and were assigned to oxo ligand titanium-bounded (fwhm = 1.7 eV) and hydroxyl groups (fwhm = 2.4 eV).

#### 3.2.7. FEG-SEM-XEDS and TEM-SAED-XEDS

The microstructure of the samples was analyzed during different steps of the reactions and before and after calcination by means of field-emission scanning electron microscopy-energy dispersive X-ray (FEG-SEM-XEDS) (Hitachi S-4700 type I, Tokyo, Japan).

Calcined powders were also characterized by transmission electron microscopy (TEM) and selected area electron diffraction (SAED) were carried out on a Jeol 2000FX (Tokyo, Japan) microscope working at 200 keV, equipped with an ISIS analyzer system that characterized the local composition by X-ray energy-dispersive spectrometry (XEDS).

## 4. Conclusions

It has been established and monitored a protocol of synthesis to obtain homogeneous Tb-doped TiO_2_ (anatase) HSs based on a simultaneous implantation of Ti and Tb species on a PST–PDVB polymer first, followed by a sol-gel process. The carbonyl groups of the copolymer template take part during the hydrolysis according to IR results. The mechanism of this process involves proton attack on the Ti–Cl, Tb–Cl and C=O bonds, the occupation of a vacant site by a water molecule and then, the dissociation of the dangling Ti–Cl, Tb–Cl ligands and C=O bonds, according to the observed decrease of both the band intensity and of the wavenumbers of the carbonyl group. Ti_1−x_Tb_x_[(OH)_4−y_Cl_z_]@PST–PDVB and Ti_1−x_Tb_x_[(OH)_4−u__′_]@PDVB intermediate complexes are formed during this step. Finally, polycondensation leads to amorphous Ti_1−x_Tb_x_O_2−w′_@PST–PDVB compounds. This synthesis route allows us to prepare TiO_2_ and Tb-doped TiO_2_ HSs with pure anatase phase structure after calcination according to XRD. XPS demonstrated the presence of Tb^4+^ with a fraction of 38.5 and 41.2% in the HSs, with 1.70 and 3.00 at.% Tb, respectively, in addition to Tb^3+^. Then, our doped HSs doped with these compositions can be considered as co-doped systems and as is observed in previous studies for co-doped RE systems the ART is favored. An increasing fraction of Tb with calcination temperature for a given composition confirmed a Tb diffusion process from the inner part to the surface of the HSs. Finally, the synthesis method described in this work could be extended to the preparation of other RE-doped anatase systems.

## Figures and Tables

**Table 1:** Specific surface areas of the HSs calcined at 550 °C.

	SSA (m^2^/g)
0.00%_550	44 ± 3
0.12%_550	35 ± 4
1.70%_550	34 ± 4
3.00%_550	32 ± 4

**Table 2:** Quantitative analysis of the surfaces for selected samples (at.%: percentage of atomic concentration).

	Tb (at. %)	Ti (at. %)	O (at. %)	O/Ti Ratio	Tb/Ti Ratio
0.00%_550	-	25.3	74.7	3.0	-
0.12%_550	0.1	27.4	72.5	2.6	0.004
1.70%_550	1.4	27.3	71.3	2.6	0.050
3.00%_550	2.7	27.9	69.4	2.5	0.100
1.70%_800	2.8	27.5	69.7	2.5	0.102
1.70%_1000	3.9	25.3	70.8	2.8	0.154

**Table 3:** Quantitative analysis of the deconvolution of Tb 3d_5/2_ signal with two components: Tb^3+^ and Tb^4+ (a)^ and quantitative analysis of the deconvolution of X-ray photoemission O 1s signal with two components ^(b)^: Ti-O lattice and Ti-OH terminal (at.%: percentage of atomic concentration).

	Tb^4+ (a)^ (at.%)	Tb^3+ (a)^ (at.%)	Tb^4+^/Tb^3+^ Ratio	Ti-O ^(b)^ Lattice (at.%)	Ti-OH ^(b)^ Terminal (at.%)
0.00%_550	-	-	-	61.5	38.5
0.12%_550	-	-	-	83.0	17.0
1.70%_550	38.5	61.5	61.5	80.7	19.3
3.00%_550	41.2	58.8	58.8	83.5	16.5
1.70%_800	43.9	56.1	56.1	83.6	16.4
1.70%_1000	49.5	50.5	50.5	83.5	16.5

**Table 4:** Anatase lattice parameters, cell volumes, and crystallite sizes of the HSs calcined at 550 °C.

	a ± 0.004 (Å)	c ± 0.005 (Å)	V ± 0.02 (Å^3^)	Crystallite Size ± 0.2 (nm)
0.00%_550	3.786	9.520	136.44	9.9
0.12%_550	3.784	9.515	136.24	12.0
1.70%_550	3.786	9.517	136.41	12.5
3.00%_550	3.786	9.518	136.46	12.8

## Data Availability

Not applicable.

## Supplementary Materials

The following supporting information can be downloaded at: https://www.mdpi.com/article/10.3390/ijms232113162/s1.
