# Supplementary material for: Monitoring the Simultaneous Implantation of Ti and Tb Cations to a Sacrificial Template and the Sol-Gel Synthesis of Tb-Doped TiO2 (Anatase) Hollow Spheres and Their Transition to Rutile Phase
Source: Int J Mol Sci. 2022 Oct 29;23(21):13162. doi: 10.3390/ijms232113162 (PMC9657842; doi:10.3390/ijms232113162)
Supplement: Supplementary file 1 [file ijms-23-13162-s001.zip › ijms-1974178-supplementary.pdf]

International Journal of
Molecular Sciences

MDPI

Supplementary materials

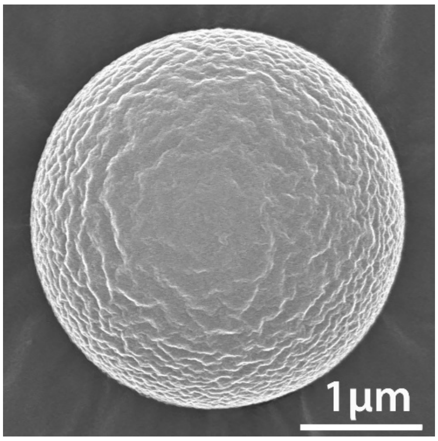

Figure S1. FEG-SEM image of the template.

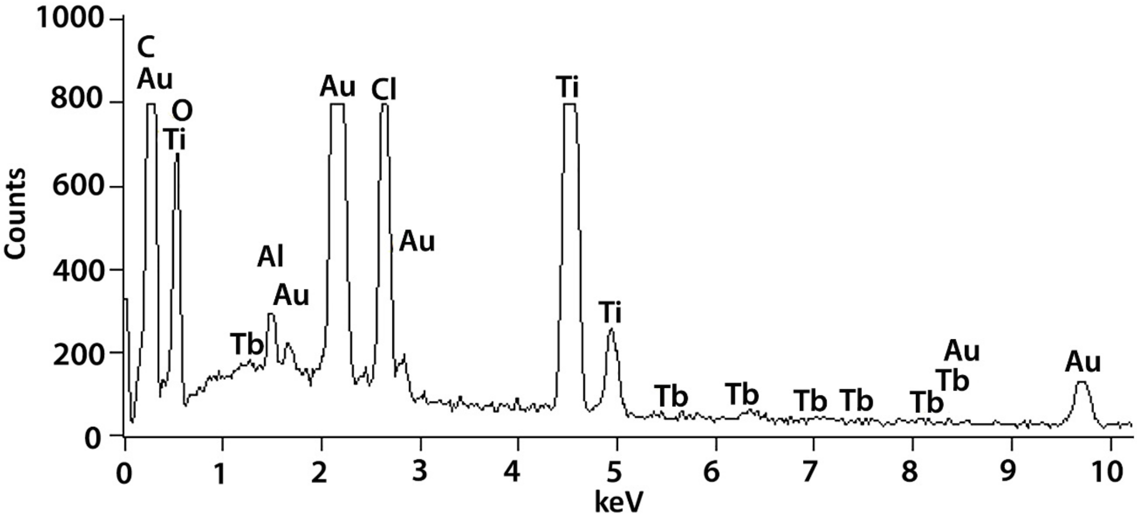

Figure S2. XEDS analysis of the template + $TiCl_4$ and $TbCl_3$ mixture + HCl (aq) after 5 h of reaction for the 0.12 at.% Tb (Fig. 4 (a) is the corresponding FEG-SEM picture of the sample).

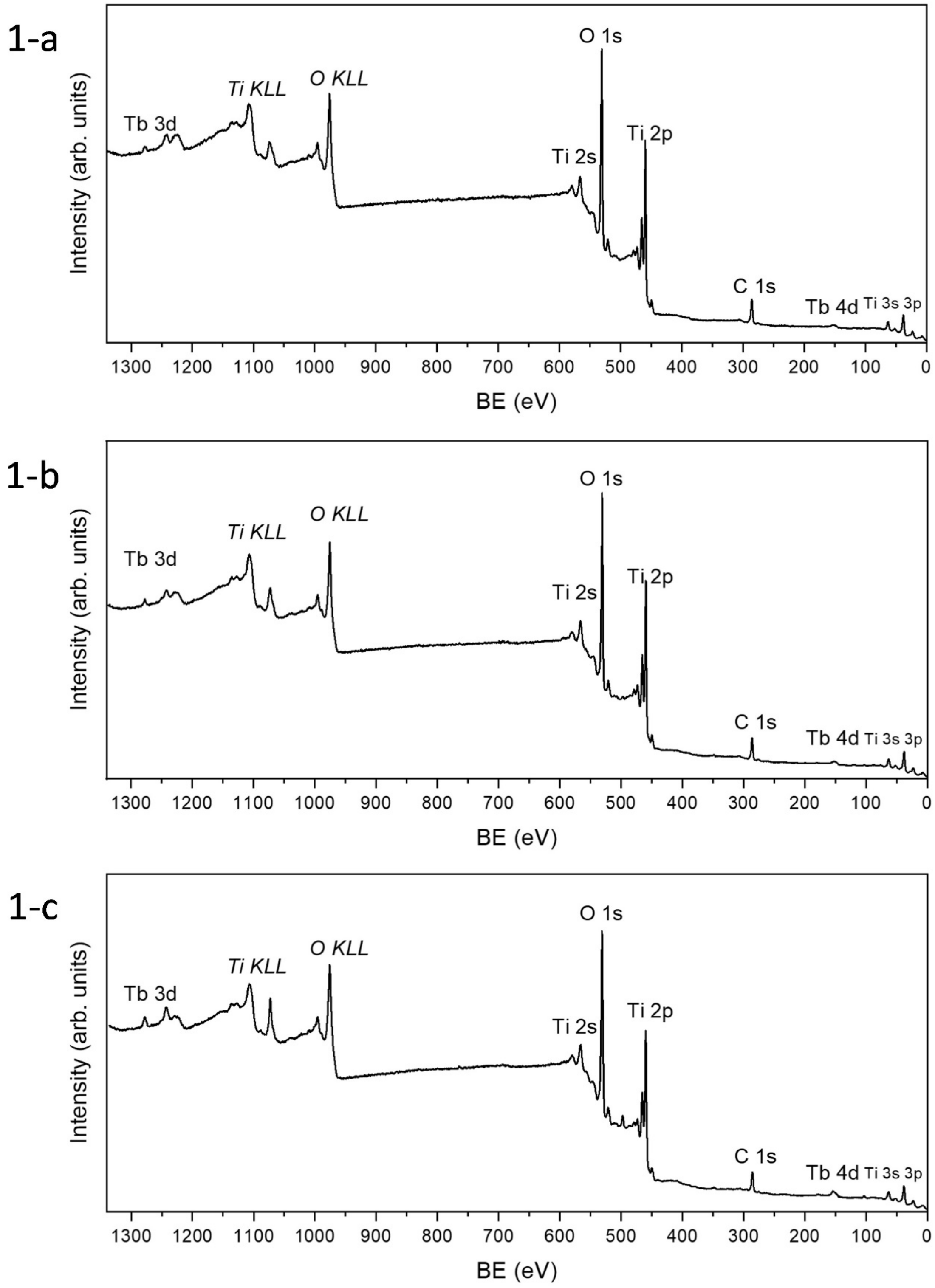
1-a
1-b
1-c
Tb 3d
Ti KLL
O KLL
O 1s
Ti 2s
Ti 2p
C 1s
Tb 4d
Ti 3s 3p
Intensity (arb. units)
BE (eV)
1300 1200 1100 1000 900 800 700 600 500 400 300 200 100 0

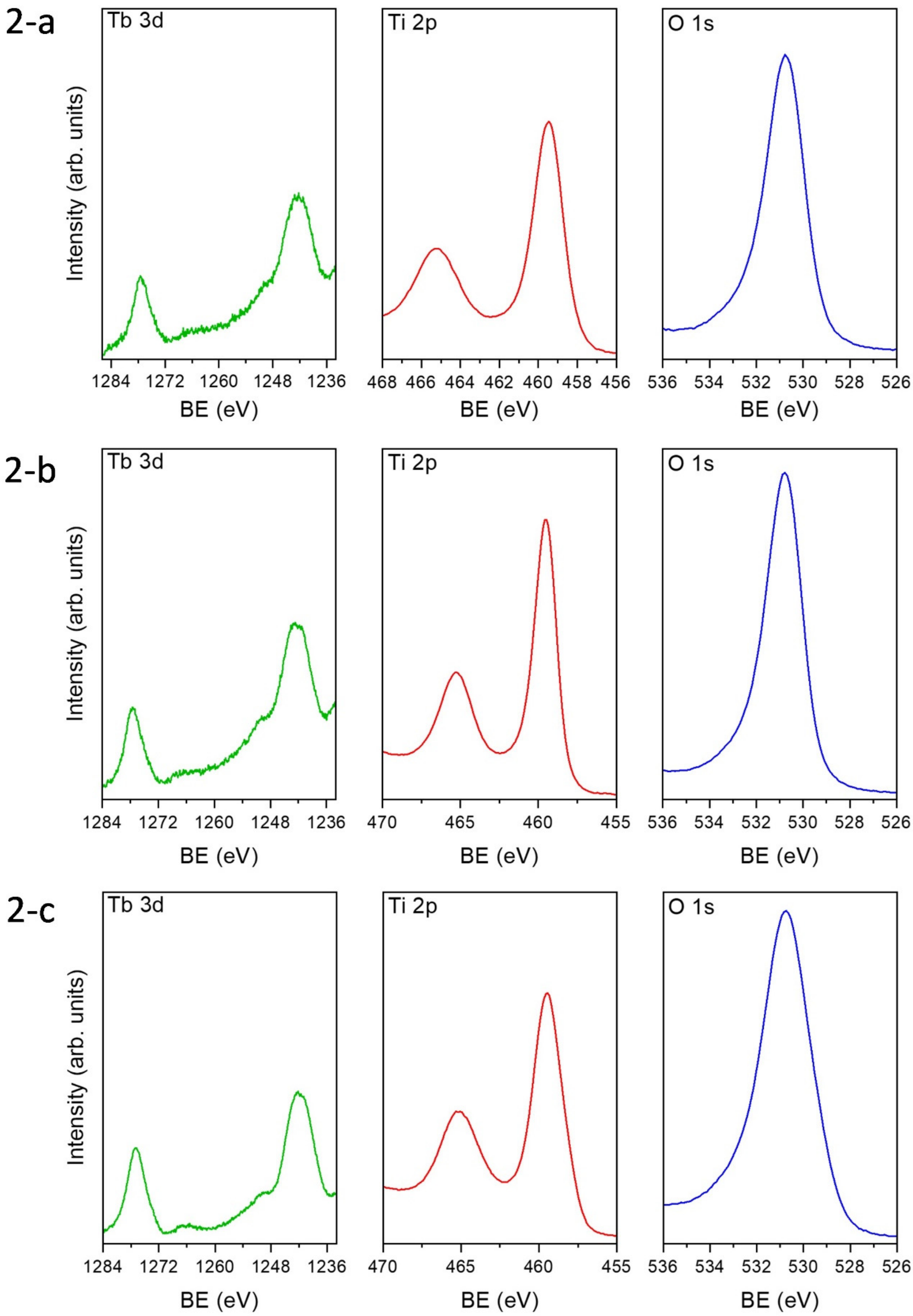

Figure S3. Survey photoemission spectra of 3.00%_550 (**1-a**), 1.70%_800 (**1-b**) and 1.70%_1000 (**1-c**), respectively. Detailed X-ray photoelectron spectral regions of the main signals for the Tb 3d, Ti 2p and O 1s elements in 3.00%_550 (**2-a**), 1.70%_800 (**2-b**) and 1.70%_1000 (**2-c**), respectively.

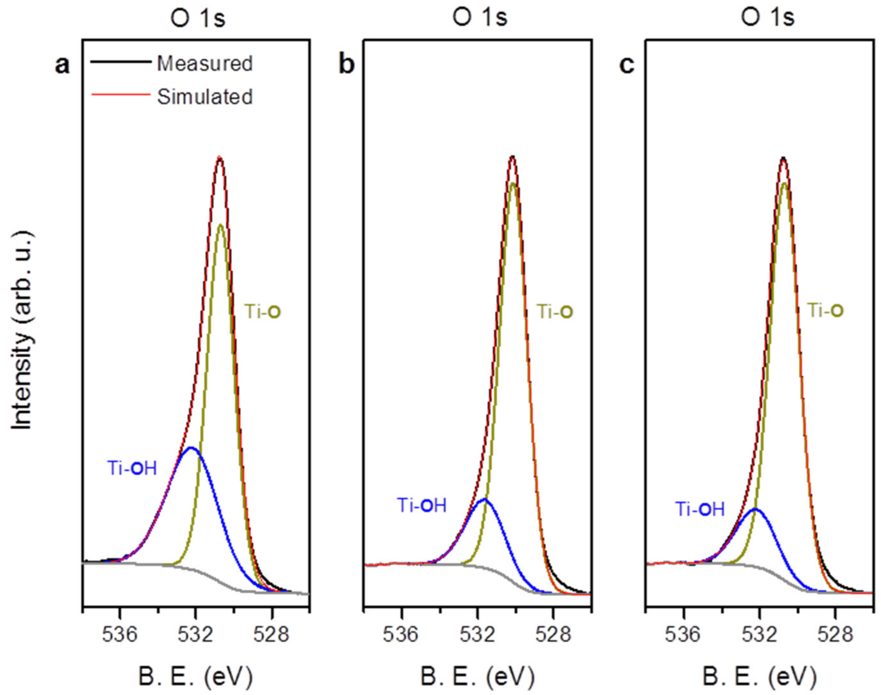

Figure S4. Experimental and fitted O 1s X-ray photoelectron spectral region of the samples calcined at 550 °C that shows two different bounded oxygen ((**a**): 0.00%_550; (**b**): 1.70%_550 and (**c**): 3.00%_550).
